# Investigation of immune response induction by Japanese encephalitis live‐attenuated and chimeric vaccines in mice

**DOI:** 10.1002/mco2.117

**Published:** 2022-04-06

**Authors:** Enyue Fang, Xinyu Liu, Xiaohui Liu, Ming Li, Ling Wang, Miao Li, Zelun Zhang, Yuhua Li, Yongxin Yu

**Affiliations:** ^1^ National Institutes for Food and Drug Control Beijing 102629 China; ^2^ Wuhan Institute of Biological Products, Co., LtD. Wuhan 430207 China

**Keywords:** chimeric vaccine, Japanese encephalitis, live‐attenuated vaccine, neutralizing antibodies, protection, T‐cell responses

## Abstract

The Japanese encephalitis (JE) live‐attenuated vaccine SA14‐14‐2 and the chimeric vaccine IMOJEV (JE‐CV) are two kinds of vaccines available for use worldwide. JE‐CV was previously known as ChimeriVax‐JE, that consists of yellow fever vaccine 17D (YFV‐17D) from which the structural genes (prM/E) have been replaced with those of SA14‐14‐2. This study aimed to investigate the neutralizing antibody, protection efficacy, and specific T‐cell response elicited by both vaccines in mice. The neutralizing antibodies produced by JE‐CV were slightly lower than those produced by SA14‐14‐2, but the protection conferred by JE‐CV was considerably lower in the low vaccine dose immunization group. Furthermore, the JE‐CV did not induce a specific T‐cell response against JEV NS3, while it did induce a potent antigen‐specific T‐cell response against the viral backbone vaccine YFV. In conclusion, this study is the first detailed investigation of the cellular immune response to the two vaccines. Enzyme‐linked immunospot (ELISPOT) and flow staining suggest a more potent specific T‐cell response against the JEV antigen was elicited in mice immunized with SA14‐14‐2 but not JE‐CV. Using heterologous flaviviruses as a live‐attenuated vaccine backbone may unlikely generate an optimal T‐cell response against the vaccine strain virus and might affect the protective efficacy.

## Graphical abstract

Live‐attenuated vaccine SA14‐14‐2 and chimeric vaccine JE‐CV are two kinds of JEV vaccines available worldwide. The satisfied immune protection relays humoral and cellular immunity. The nonstructural proteins of SA14‐14‐2 can elicit robust T‐cell response, but JE‐CV lack the nonstructural proteins of JEV. Therefore, using heterologous flaviviruses as backbone is unlikely to induce optimal T‐cell response against the vaccine virus.

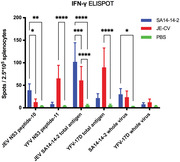

## 1 INTRODUCTION

Flaviviruses, such as the yellow fever virus (YFV), Zika virus (ZIKV), dengue virus (DENV), and Japanese encephalitis virus (JEV), are pathogens transmitted by mosquito vectors. In recent years, this class of viruses have been rapidly disseminated globally. JEV is present in 24 Southeast Asian and Western Pacific nations, with an annual estimate of 68,000 cases and over three billion individuals at risk of infection.^1^, ^2^, ^3^ In general, one of the most effective and safe ways to prevent the transmission of the virus is vaccination, which is now the case for the world pandemic SARS‐CoV‐2,^4^ and likewise for JEV. Therefore, it is necessary to strengthen the research and development of vaccines.

For worldwide immunization, there are three classes of Japanese encephalitis (JE) vaccines available, which include the JE live‐attenuated vaccine SA14‐14‐2, the chimeric vaccine IMOJEV (JE‐CV), previously known as ChimeriVax‐JE, and the SA14‐14‐2 inactivated Vero cell‐based vaccine.^5^, ^6^, ^7^ The World Health Organization (WHO) has prequalified and authorized the usage of the vaccine SA14‐14‐2 in more than 12 Asian countries.^8^, ^9^ JE‐CV is constructed with prM and E antigen genes of SA14‐14‐2 using the live‐attenuated virus YFV‐17D as the backbone. JE‐CV has been authorized for commercial use by the WHO.^6^ Both vaccines were found to be safe and they produced strong neutralizing antibody responses in humans.^5^, ^6^, ^10^, ^11^, ^12^, ^13^, ^14^ However, vaccination responses with high protective effectiveness have only been recorded in clinical investigations with the vaccine SA14‐14‐2,^15^, ^16^, ^17^, ^18^, ^19^ whereas there are currently no reports on the efficacy of the vaccine JE‐CV.

In clinical trials, a tetravalent dengue chimeric vaccine Dengvaxia (CYD‐TDV) using the same technology was authorized for human use, excluding children less than 9 years of age or naive individuals, since there were more severe DENV infections in these subpopulations.^20^, ^21^, ^22^, ^23^, ^24^, ^25^ The lack of the dengue virus nonstructural protein gene, which cannot produce a viral‐specific response by T cells, has been postulated as the cause.^26^, ^27^, ^28^ Recent studies have demonstrated that flavivirus nonstructural proteins, particularly nonstructural protein 3 (NS3) and nonstructural protein 1 (NS1), play a vital role in vaccination‐induced immunity.^29^, ^30^, ^31^, ^32^, ^33^, ^34^ To establish a foundation for investigating the efficacy of the JEV vaccine, we assessed the immune responses (humoral and cellular) in mice following immunization with the vaccines SA14‐14‐2 and JE‐CV, particularly the specific T‐cell responses elicited against NS3.

## 2 RESULTS

### 2.1 Neutralizing antibody titers induced in the different immunization groups at varying vaccine doses of the vaccines

**FIGURE 1:**
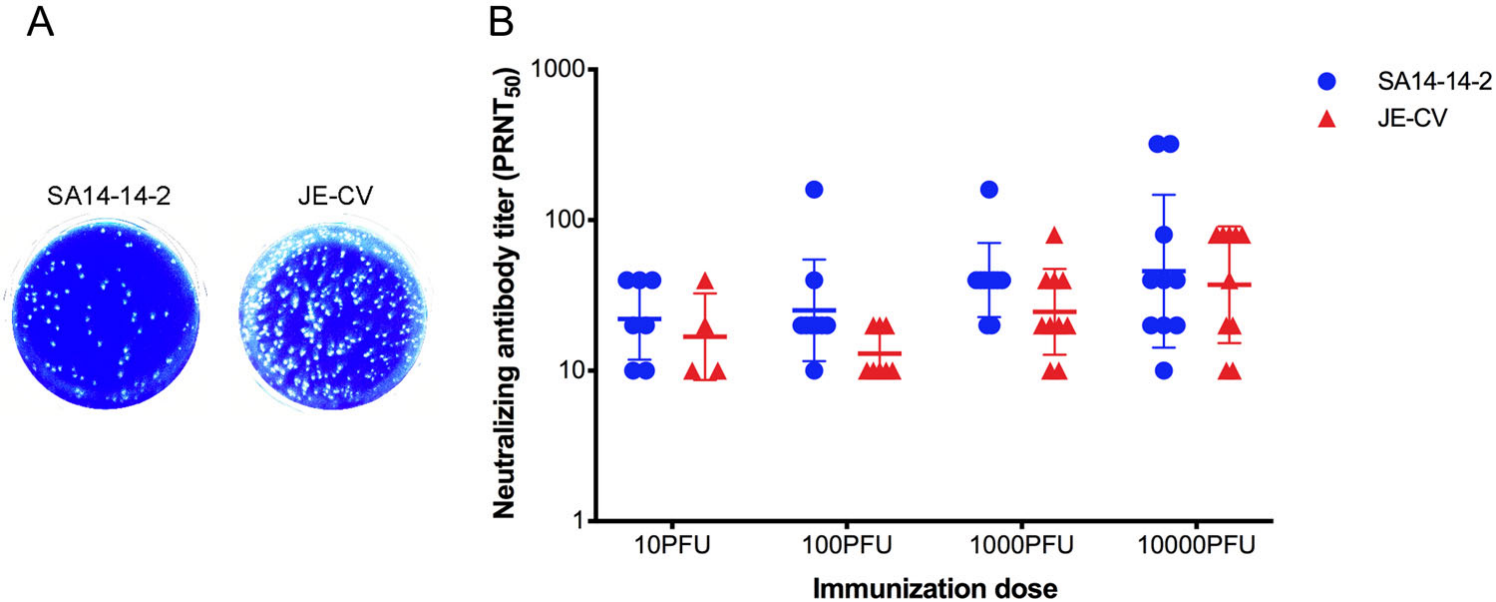
Plaque morphology and immunogenicity of the vaccines JE‐CV and SA14‐14‐2. (A) Plaque morphology of the vaccines JE‐CV and SA14‐14‐2 on BHK‐21 cells infected with serial dilutions of viruses in 10 folds. (B) Immunogenicity of the vaccines JE‐CV and SA14‐14‐2 in mice. The abscissa represents four immunization dose groups with 10 mice in each group

To evaluate the humoral immune response induced by live‐attenuated vaccine SA14‐14‐2 and the chimeric vaccine JE‐CV, sera samples in mice were collected and the plaque reduction neutralization testing (PRNT) assay was applied to detect the titers of the anti‐JEV neutralizing antibody at 2 weeks after vaccination at varying doses of the vaccines. The geometric mean titers (GMTs) of neutralizing antibodies in mice vaccinated with 10, 100, 1000, and 10,000 PFU doses of SA14‐14‐2 were 22, 25, 40, and 46, respectively, and the seroconversion rates were 70%, 90%, 100%, and 100%, respectively. The GMTs induced by mice vaccinated with a constant dose of JE‐CV were 17, 13, 25, and 37, and the seroconversion rates were 40%, 80%, 100%, and 100%, respectively (Table 1). At the same immunization dose, the neutralizing antibody of SA14‐14‐2 vaccine group was slightly higher than JE‐CV vaccine. There were no significant differences among the groups vaccinated at a constant dose (Figure 1).

**TABLE 1:** Neutralizing antibody and protective efficacy of the vaccines JE‐CV and SA14‐14‐2

Immunogen	Immunization dose (PFU)	Neutralizing antibody titer (GMT)^a^	Seroconversion rate^b^	Survival rate^c^
SA14‐14‐2	10	22	7/10	8/10
	100	25	9/10	10/10
	1000	40	10/10	10/10
	10,000	46	10/10	10/10
JE‐CV	10	17	4/10	2/10
	100	13	8/10	8/10
	1000	25	10/10	9/10
	10,000	37	10/10	10/10
PBS	Not applicable	Not applicable	0/10	0/10

^a^ Titers of <10 were not included in the calculation of geometric mean titer (GMT).

^b^ No. seroconversion/no. tested. PRNT_50_ titer ≥ 1:10 was considered seroconversion.^35^, ^36^, ^37^, ^38^

^c^ No. survive/No. total.

### 2.2 Protection efficacy in mice after challenge

**FIGURE 2:**
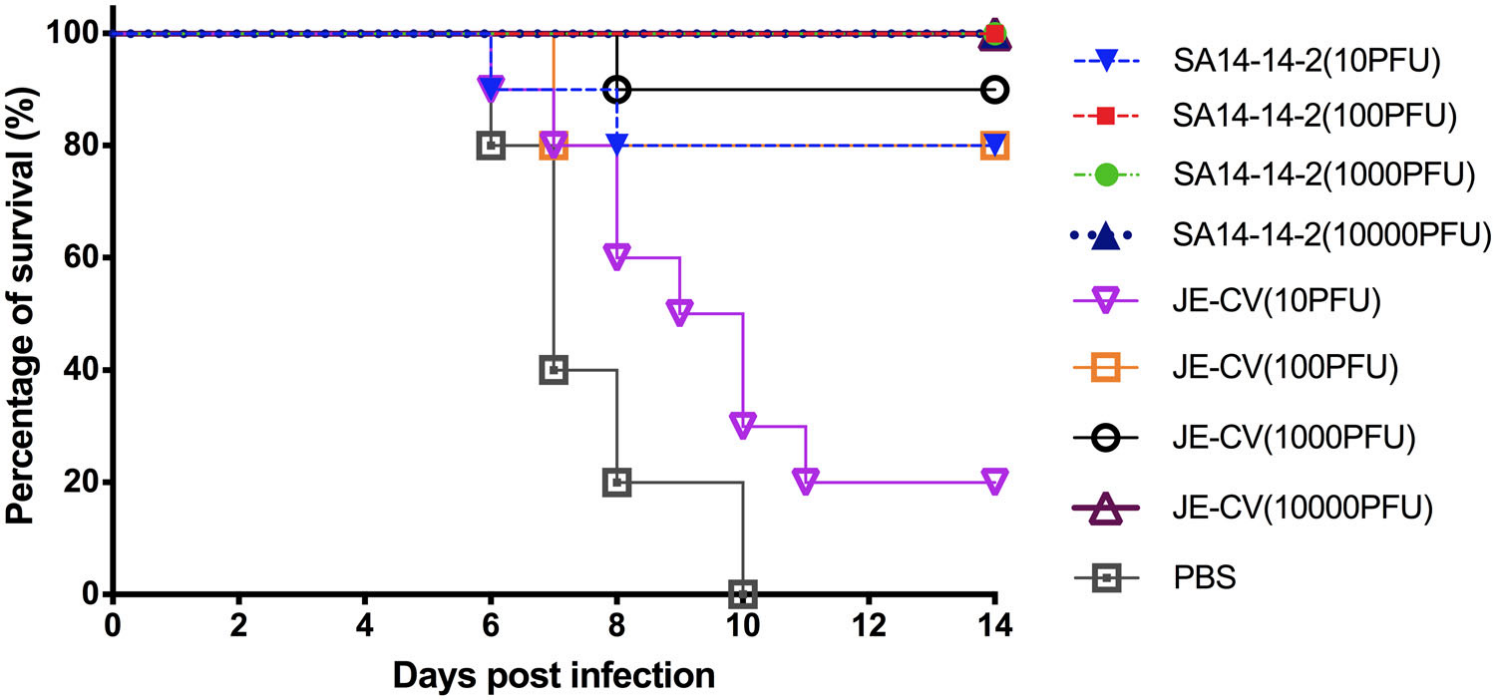
Survival data of mice immunized with different doses of SA14‐14‐2 and JE‐CV following administration of a lethal dose (3000 LD_50_) of JEV P3 strain (*n* = 10)

As live virus vaccines can replicate and amplify after entering the host, low‐dose immunization can also be protective. To determine the protective efficacy of both JE‐CV and SA14‐14‐2 vaccines in mice, experiments were performed using four different immunization doses, and the JEV P3 strain was challenged intraperitoneally at 14 days after immunization. The results showed that mice immunized with high doses (100, 1000, and 10,000 PFU) of the vaccine SA14‐14‐2 had 100% protection, and those immunized with high doses of the vaccine JE‐CV had similar high protection. However, mice immunized with a low dose (10 PFU) of the vaccine SA14‐14‐2 had 80% protection, whereas those immunized with a low dose of the vaccine JE‐CV had only 20% protection, thus showing statistical significance (*p *< 0.05) (Figure 2).

### 2.3 Identification and screening of T‐cell epitopes of JEV and YFV NS3 proteins

**FIGURE 3:**
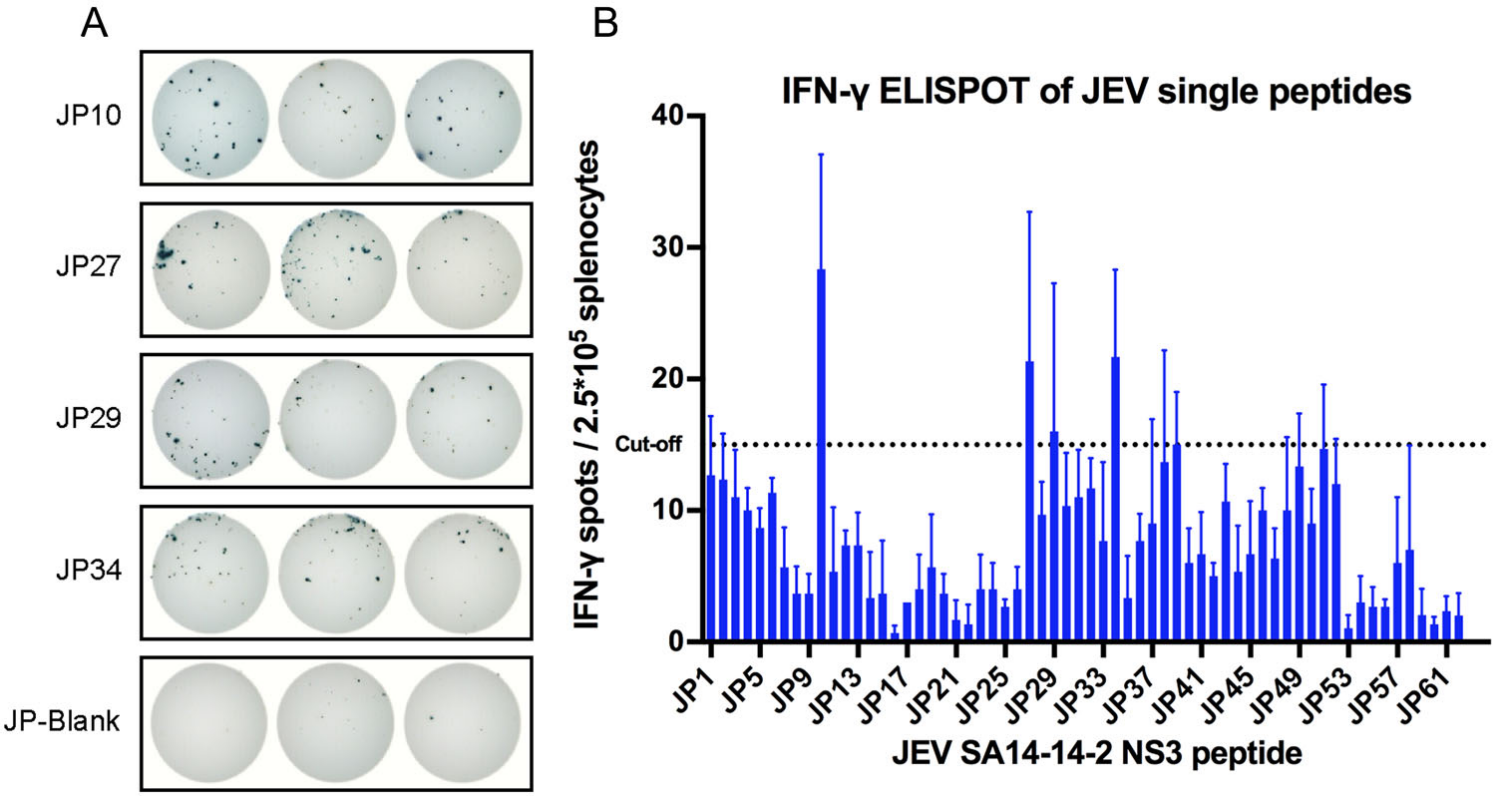
JEV NS3 single‐peptide screening. Four‐week‐old BALB/c mice were injected subcutaneously with 10^4^ PFU SA14‐14‐2. Splenocyte‐derived cytokine levels were detected through IFN‐γ enzyme‐linked immunospot (ELISPOT) assay with JEV NS3 single‐peptide stimulus on day 28 after infection. (A) The spots of splenocytes for the positive peptides or negative control. Experiments were set up with three replicate wells. (B) The IFN‐γ ELISPOT of JEV single peptides. The cytokine‐positive cell count is expressed as SFU/2.5 × 10^5^ cells. Each column of the abscissa corresponds to a peptide. The cutoff value (15 SFU) corresponds to three times the background obtained with unstimulated cells

**FIGURE 4:**
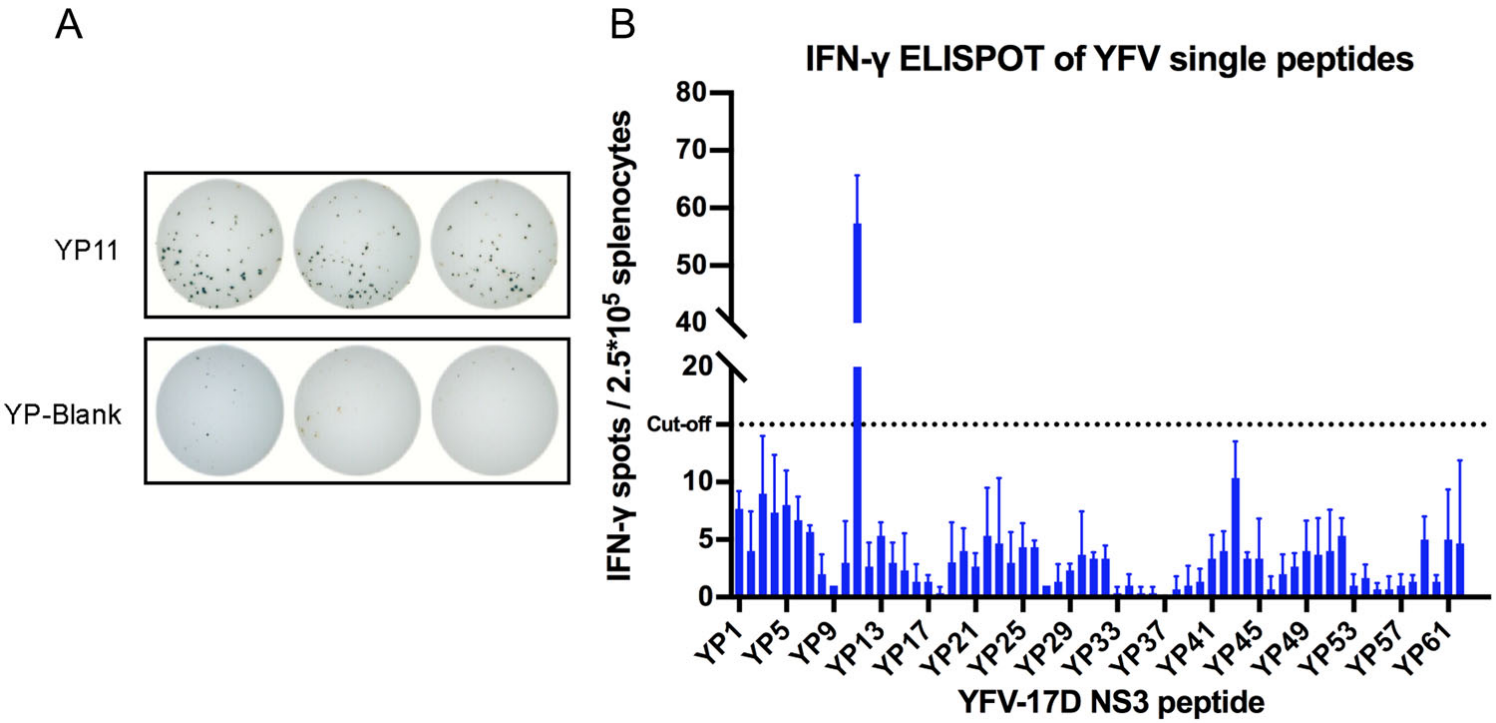
YFV NS3 single‐peptide screening. Four‐week‐old BALB/c mice were injected subcutaneously with 10^4^ PFU YFV‐17D. Splenocyte‐derived cytokine levels were detected through IFN‐γ enzyme‐linked immunospot (ELISPOT) assay with YFV NS3 single‐peptide stimulus on day 28 after infection. (A) The spots of splenocytes for the positive peptides or the negative control. Experiments were set up with three replicate wells. (B) The IFN‐γ ELISPOT of YFV single peptides. The cytokine‐positive cell count is expressed as SFU/2.5 × 10^5^ cells. Each column of the abscissa corresponds to a peptide. The cutoff value (15 SFU) corresponds to three times the background obtained with unstimulated cells

To determine the stimulators of specific T‐cell epitope antigens for cellular immunity experiments, we selected 62 pieces of overlapping peptides from the NS3 proteins of JEV and YFV, respectively. Each of these overlapping peptides contains 15 amino acids, with one peptide at 10 amino acids intervals. BALB/c mice were vaccinated with YFV‐17D and SA14‐14‐2, respectively, and a single peptide was used as a stimulator to detect the secretion of interferon‐gamma (IFN‐γ) by mouse splenocytes after 4 weeks. A positive stimulator was determined when the spot‐forming units (SFUs) in the stimulated group were three times higher than those in the nonstimulated group. The enzyme‐linked immunospot (ELISPOT) assay revealed that the secretion of IFN‐γ after stimulation of JP10 (****p < 0.0001), JP27 (****p < 0.0001), JP29 (**p < 0.01), and JP34 (****p < 0.0001) in the JEV overlapping peptide pool was significantly different compared to the immunized but nonstimulated group, with the highest secretion of 113 SFU/million splenocytes in JP10 (Figure 3; Table 2). YP11 in the YFV overlapping peptide pool showed a statistically significant difference from the immunized but nonstimulated group (****p < 0.0001), with a secretion of 229 SFU/million splenocytes, which was more than that of JP10 (Figure 4; Table 2).

**TABLE 2:** Enzyme‐linked immunospot (ELISPOT) responses to the immunodominant JEV‐ and YFV NS3‐specific peptides

Vaccine	No. of peptides	Sequence	Origin of sequences	Location (amino acid)	IFN‐γ spots/million splenocytes (mean ± SD)
SA14‐14‐2	JP10	GTDDVQVIVVEPGKG	SA14‐14‐2	NS3 (91–105)	113 ± 20^****^
	JP27	MCHATLTHRLMSPNR		NS3 (261–275)	85 ± 26^****^
	JP29	LFVMDEAHFTDPASI		NS3 (281–295)	64 ± 26^**^
	JP34	APIHDLQDEIPDRAW		NS3 (331–345)	87 ± 15^****^
YFV‐17D	YP11	AAVPGKNVVNVQTKP	YFV‐17D	NS3 (101–115)	229 ± 19^****^

### 2.4 SA14‐14‐2 induces T‐cell response against JEV‐NS3 peptide but not JE‐CV

**FIGURE 5:**
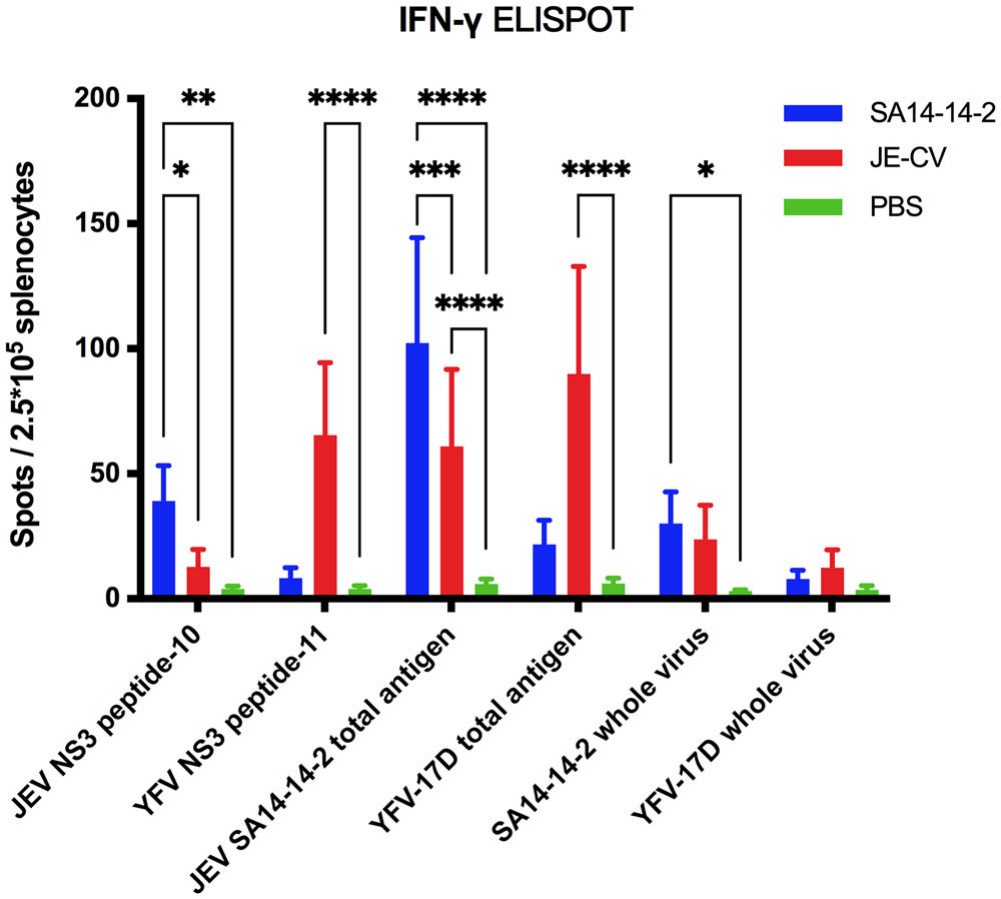
Cell‐mediated immune responses were elicited in 4‐week‐old BALB/c mice 4 weeks after immunization by a single injection of 10^4^ PFU of JE‐CV and SA14‐14‐2, respectively. Splenocytes were stimulated with JEV NS3 peptide, YFV NS3 peptide, JEV total antigen, YFV total antigen, JEV whole virus, and YFV whole virus, respectively. Secretion of cytokines was detected by enzyme‐linked immunospot (ELISPOT) (*n* = 6). After background subtraction, the cytokine‐positive cell counts were expressed as SFU/2.5 × 10^5^ cells. Data were obtained from two independent experiments. Statistical significance was determined by two‐way analysis of variance (ANOVA; *p < 0.05, **p < 0.01, ***p < 0.001, ****p < 0.0001)

**FIGURE 6:**
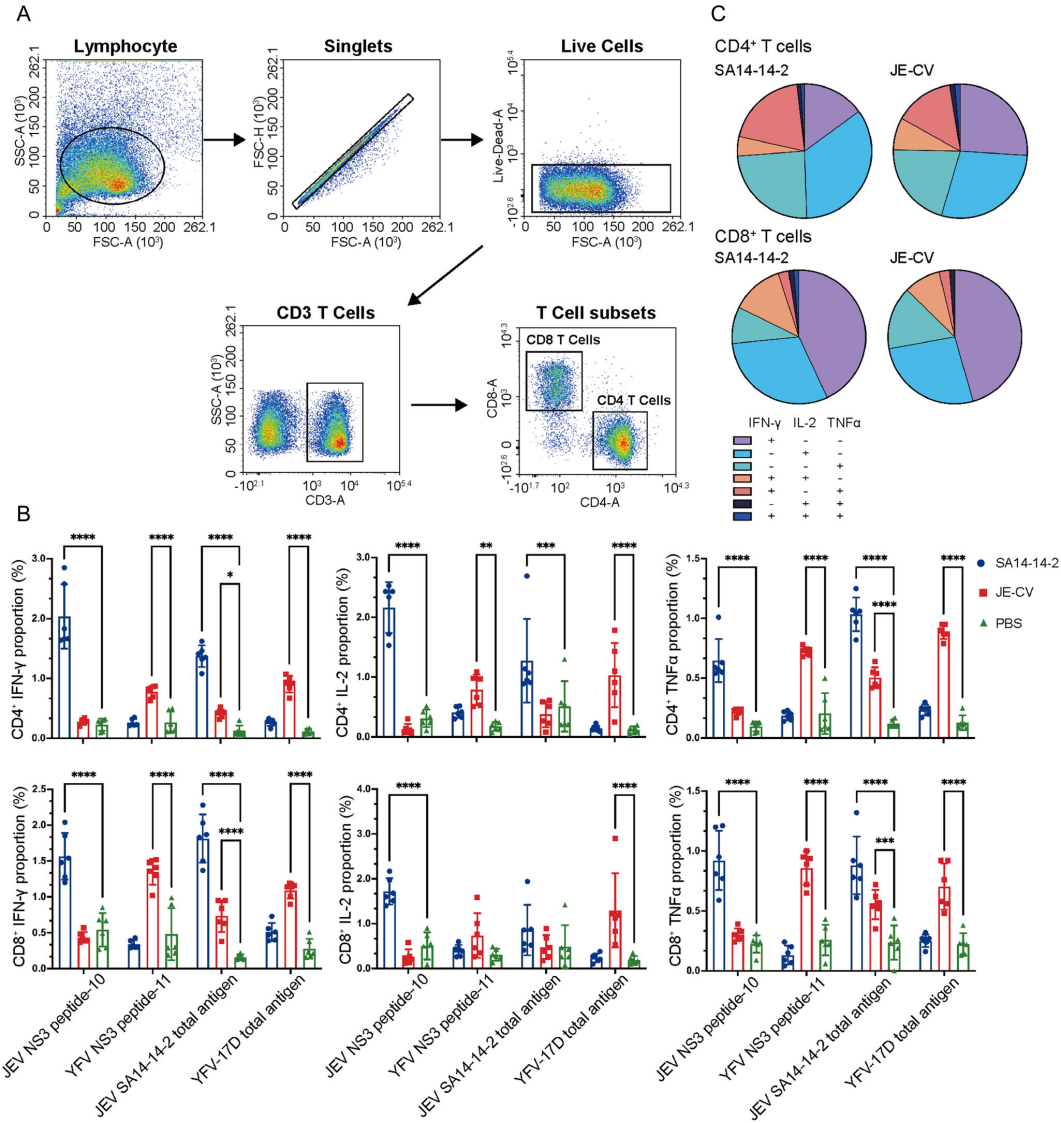
Flow cytometry of intracellular cytokine staining after vaccination. The IFN‐γ, IL‐2, and TNF‐α production profiles of JEV‐ and YFV‐specific CD4^+^ and CD8^+^ T cells in BALB/c mice immunized with SA14‐14‐2 and JE‐CV 4 weeks after immunization are shown. Splenocytes were stimulated with JEV and YFV NS3 peptides and with lysates of SA14‐14‐2‐infected and YFV‐17D‐infected Vero cells (8 h ex vivo). (A) Gating strategy. Lymphocytes were first gated. Single cells were gated according to FSC‐A versus FSC‐H. T cells were gated as CD3^+^ population in live cells. T‐cell subsets of CD4^+^CD8^–^ and CD4^–^CD8^+^ were gated as CD4^+^ T and CD8^+^ T cells. Finally, the expressions of IFN‐γ, IL‐2, and TNF‐α in these two T‐cell populations were analyzed. (B) Percentage of cells secreting IL‐2, IFN‐γ, and TNF‐α from CD4^+^ T or CD8^+^ T cells. (C) Percentage of CD4^+^ T and CD8^+^ T cells producing any combination of IL‐2, IFN‐γ, and TNF‐α stimulated with cell lysate of SA14‐14‐2‐infected Vero cells. Data were obtained from two independent experiments. Statistical significance was determined by two‐way ANOVA (*p < 0.05, **p < 0.01, ***p < 0.001, ****p < 0.0001)

We evaluated the T‐cell‐mediated immune response induced by the vaccines SA14‐14‐2 and JE‐CV against JEV nonstructural proteins. Four weeks after immunization, levels of the splenocyte‐derived cytokines were determined in each group (Figures 5 and 6). According to ELISPOT results, the level of IFN‐γ in the SA14‐14‐2 group stimulated with JEV NS3 peptide‐10 (JP10) was significantly increased (**p < 0.01), whereas the level in the JE‐CV group was nonsignificantly different from that in the control group. On the other hand, stimulation with YFV NS3 peptide‐11 (YP11) resulted in a significant increase in IFN‐γ level in the JE‐CV group compared to the control group, whereas the level was nonsignificantly increased in the SA14‐14‐2 group (Figure 5).

The flow cytometry‐based gate strategy analysis was performed as described in Figure 6A. The results of flow cytometry showed that both CD4^+^ IFN‐γ^+^ (****p < 0.0001) and CD8^+^ IFN‐γ^+^ cell counts (****p < 0.0001) were significantly increased in the SA14‐14‐2 group, but not in the JE‐CV group, after stimulation with JP10, as compared to the control group. In contrast, after stimulation with YP11, the CD4^+^ IFN‐γ^+^ (****p < 0.0001) and CD8^+^ IFN‐γ^+^ cell counts (****p < 0.0001) were significantly increased in the JE‐CV group, but not in the SA14‐14‐2 group, as compared to the control group. Similar results were observed in CD4^+^ TNFα^+^, CD8^+^ TNFα^+^, CD4^+^ IL‐2^+^, and CD8^+^ IL‐2^+^ cells counts. However, CD8^+^ IL2^+^ cells count in the JE‐CV group was nonsignificantly increased after stimulation with YP11 (Figure 6B).

### 2.5 SA14‐14‐2 induces higher T‐cell responses than JE‐CV against the total antigen of JEV cell lysates and whole‐virus stimulations

To compare the difference in the cellular immune response induced by the total antigen of JEV after immunization with SA14‐14‐2 and JE‐CV, the total antigen from cell lysates of JEV and YFV and the cell supernatant from the infected virus were used as stimulants to detect the splenocyte‐derived cytokine levels. The ELISPOT assay showed significantly increased levels of IFN‐γ in the SA14‐14‐2 (****p < 0.0001) and JE‐CV (****p < 0.0001) groups after stimulation with JEV total antigen compared to the control group, respectively, and that the SA14‐2 group induced significantly higher levels of IFN‐γ than the JE‐CV group (***p < 0.001). After stimulation with SA14‐14‐2 viral supernatant, IFN‐γ response was significantly higher in the SA14‐14‐2 group compared with the control group (*p < 0.05); however, there was a nonsignificant increase in IFN‐γ response in the JE‐CV group compared with the control group. In contrast, after stimulation with the total antigen from YFV‐17D cell lysates, IFN‐γ response was significantly increased in the JE‐CV group than in the control group (****p < 0.0001), whereas it was nonsignificantly increased in the SA14‐14‐2 group. After stimulation with YFV‐17D viral supernatant, IFN‐γ response in both SA14‐14‐2 and JE‐CV groups was nonsignificantly increased (Figure 5).

After stimulation of total antigen from JEV SA14‐14‐2 cell lysates, flow cytometry revealed that the CD4^+^ IL‐2^+^ (***p < 0.001), CD4^+^ IFN‐γ^+^ (****p < 0.0001), CD4^+^ TNFα^+^ (****p < 0.0001), CD8^+^ IFN‐γ^+^ (****p < 0.0001), and CD8^+^ TNF α^+^ (****p < 0.0001) cells counts were significantly higher in the SA14‐14‐2 group compared to the control group. The CD4^+^ IFN‐γ^+^ (*p < 0.05), CD4^+^ TNFα^+^(****p < 0.0001), CD8^+^ IFN‐γ^+^(****p < 0.0001), and CD8^+^ TNFα^+^(***p < 0.001) cells counts were also significantly higher in the JE‐CV group compared to the control group (Figure 6B). Furthermore, the counts of these aforementioned cells were higher in the SA14‐14‐2 group than in the JE‐CV group (Figure 6B); however, CD8^+^ IL‐2^+^ cells counts were not significantly increased in both the SA14‐14‐2 and JE‐CV groups compared with the control group (Figure 6B). In contrast, after stimulation with YFV‐17D cell lysate total antigen, the CD4^+^ IL‐2^+^ (****p < 0.0001), CD4^+^ IFN‐γ^+^ (****p < 0.0001), CD4^+^ TNFα^+^ (****p < 0.0001), CD8^+^ IFN‐γ^+^ (****p < 0.0001), CD8^+^ IL‐2^+^ (****p < 0.0001), and CD8^+^ TNFα^+^ (****p < 0.0001) cells counts were significantly higher in the JE‐CV group compared to the control group, whereas these cell counts were nonsignificantly increased in the SA14‐14‐2 group (Figure 6B).

The levels of cytokines induced by CD4^+^ T and CD8^+^ T cells were similar in both immunized groups stimulated in vitro with the total antigen from JEV cell lysates. The active CD4^+^ T cells mainly secrete IL‐2, and a higher CD4^+^ T‐cell proportion could secrete both IFN‐γ and TNFα. In contrast, the active CD8^+^ T cells mainly secrete IFN‐γ, and a higher CD8^+^ T‐cell proportion could secrete both IFN‐γ and IL‐2. The CD4^+^ T‐ or CD8^+^ T‐cell proportion that secretes IFN‐γ, IL‐2, and TNFα is limited (Figure 6C).

## 3 DISCUSSION

Since 1989, the vaccine SA14‐14‐2 (JE live‐attenuated) has been utilized in India, China, Korea, Nepal, Thailand, and other Asian countries; however, it was not until 2013 that the vaccine has been prequalified by WHO. Many clinical trials have demonstrated that the vaccine SA14‐14‐2 is safe and that it shows good immunogenicity.^39^, ^40^, ^41^, ^42^ The YFV‐JEV chimeric virus vaccine JE‐CV is produced by replacing the prM and E protein genes of YFV‐17D with the corresponding genes of the vaccine SA14‐14‐2 by reverse genetic techniques. Preclinical^43^, ^44^ and clinical^45^, ^46^, ^47^, ^48^ studies have reported that the vaccine JE‐CV shows good safety and seroconversion. Moreover, it has been approved for use by the WHO, the United States, and several other countries.^49^

However, in a 3‐year long‐term clinical observation, a tetravalent YFV/DENV chimeric vaccine Dengvaxia using the same technology was shown to produce low antibodies.^50^, ^51^ It is a common concern that flavivirus nonstructural proteins may play a crucial role in vaccination‐induced immunity. Thus, many experts conceived that the no response of the T cells to dengue virus nonstructural protein in the YFV/DENV chimeric vaccine might influence the immune effectiveness.^21^, ^27^, ^28^, ^52^, ^53^ As a result, this study evaluated the humoral and cellular immunity elicited by two kinds of JE live‐attenuated and chimeric vaccines and their level of specific T‐cell response elicitation against the target antigen JEV nonstructural proteins.

The protective effect of the challenge in mice was determined by a combination of neutralizing antibodies production and cellular immune induction, although, to some extent, this effect often corresponds to the level of the neutralizing antibodies produced. To assess their protective effects, we administered the two vaccines at varying viral immunization dosages. As the two vaccines are live virus‐based vaccines, the viruses can replicate after entering the mice, so that a high and sustained immune response can be elicited even after immunization with low doses of the vaccines, which is the advantage of live‐attenuated vaccines. We administered a lower viral dose (500 LD_50_) of the JEV P3 strain to mice in our previous experiments; unfortunately, the low‐dose immunization group still had better protection and there was no significant difference between the two vaccines. For this reason, a high viral dose (3000 LD_50_) was administered to mice at different immunization doses and the difference in protection between the two vaccines was observed in the low‐dose group; in this group, the neutralizing antibody and protective effects of the vaccine SA14‐14‐2 were superior to those of the vaccine JE‐CV. In our earlier research, we discovered that the neutralizing antibodies generated after one dose of SA14‐14‐2 and those generated after two shots of the inactivated vaccine are identical; however, the vaccine SA14‐14‐2 (live‐attenuated) showed better protective effects, which is a significant improvement compared to inactivated vaccines.^54^ In guinea pigs, no seroconversion was detected after immunization with vaccine SA14‐14‐2; however, viremia was completely inhibited.^55^ This indicates that the protective effect of the vaccine SA14‐14‐2 depends not only on humoral immunity but on the induction of cellular immune response.

To evaluate the specific T‐cell responses of the two vaccines, we used three forms of antigen stimulants to activate the specific T‐cell immunity. These three antigen forms included the inactivated whole virus, total antigen of cell lysate following virus infection, and peptides. The findings demonstrate that the inactivated JEV T‐cell epitope is primarily the surface virus structural protein E and that this protein cannot elicit a robust and specific cellular response to the two vaccines. The virus structural and nonstructural proteins were found in the cell lysate. Both vaccines may induce a robust and specific T‐cell response after stimulation with JEV cell lysate, although the vaccine SA14‐14‐2 is much potent than the vaccine JE‐CV. Several studies have shown that the nonstructural proteins of JEV or other flaviviruses can induce protective immunity in mice.^29^, ^56^ Mice immunized with the JEV NS1 protein showed protection against a deadly dose of the virus.^30^ Kumar et al. found that the T‐cells produced as a result of the natural infection of JEV mainly target the viral NS3 protein rather than the structural protein.^31^ Turtle et al. identified JEV‐positive peptides mainly in the NS3 protein through ELISPOT assays, although fewer amounts of these peptides were identified in the structural protein E.^32^ Roth et al. designed mRNA‐based vaccines against the dengue virus epitopes NS3, NS4B, and NS5, inducing specific T‐cell responses and protecting human HLA class I transgenic mice.^57^ Although no neutralizing antibodies were found after immunizing mice and guinea pigs with the expressed JEV NS1 antigen, our previous study revealed that it substantially protected the animals from the viral challenge and death or viremia.^58^

Furthermore, transferring spleen cells from animals immunized with live‐attenuated JE vaccine to recipients can provide significant protection against JEV infection.^59^, ^60^ We found that the T‐cell response produced by the vaccine SA14‐14‐2 has a significant protective effect and that it selectively targets the NS3 protein. Our study first synthesized the overlapping peptides library of JEV NS3 protein. Following the screening of each overlapping peptide, we acquired and used the positive peptides against JEV as stimuli to evaluate the specific T‐cell responses of the two vaccines against JEV and YFV nonstructural proteins. The results demonstrate that the JEV NS3 peptide may induce IFN‐γ secretion in mice immunized with the vaccine SA14‐14‐2 but not the vaccine JE‐CV. YFV NS3 peptide, on the other hand, may stimulate the production of IFN‐γ in mice immunized with the vaccine JE‐CV but not the vaccine SA14‐14‐2, thus suggesting that nonstructural proteins play a role in immune protection and that JE‐CV vaccination without JEV nonstructural proteins may not generate an optimal T‐cell response. Similar studies have found that the YFV/JEV chimeric vaccine can elicit a specific T‐cell response against the backbone virus YFV and that the cellular immune response against YFV antigen is significantly higher than that of JEV using viral total antigen stimulants.^61^ Other chimeric viruses showed similar outcomes.^62^ YFV/ZIKV chimeric viruses constructed by Kum et al. elicited significantly higher immune responses against the NS3 peptide or total antigen of the backbone virus YFV than the ZIKV antigen.^63^ Our results are consistent with the above findings.

The antiviral immune response relies heavily on Th1 type cellular immunity. The IFN‐γ, IL‐2, and TNF cytokine expressions in CD4^+^ and CD8^+^ T cells were also identified in this study. According to ELISPOT results, the JE‐CV chimeric vaccine can only cause the induction of a specific Th1‐type cellular response against the NS3 peptide of the backbone virus YFV but not against the NS3 peptide of JEV, which is consistent with the results of the previous JEV/DENV chimeric vaccination.^64^ The JEV/DENV‐immunized mice can only induce a Th1‐type cellular response against the backbone virus YFV. Although the genomes of DENV, ZIKV, JEV, WNV, and YFV are substantially similar, it was found that cross‐reactive T‐cell responses across these five flaviviruses are quite restricted.^65^ The nonstructural proteins of JEV play a vital role in vaccine‐induced memory T‐cell response and the nonstructural proteins of the skeleton virus mostly account for the T‐cell responses elicited by chimeric viruses.^66^

## 4 CONCLUSIONS

In this study, the immune response and protection generated by the vaccine SA14‐14‐2 (live‐attenuated) and the chimeric vaccine JE‐CV in mice were investigated. Mice immunized with the same virus dosage of the vaccines JE‐CV and SA14‐14‐2 generated slightly higher neutralizing antibodies in the SA14‐14‐2 vaccine group than that in the JE‐CV vaccine group. Moreover, in the low‐dose immunizing group, the protection conferred by the vaccine SA14‐14‐2 was considerably higher than that of the vaccine JE‐CV. A more potent specific T‐cell response against the JEV‐NS3 peptide was elicited in mice immunized with the vaccine SA14‐14‐2 but not the vaccine JE‐CV; additionally, the vaccine SA14‐14‐2 elicited greater T‐cell responses against the total antigen of JEV cell lysates and the inactivated whole‐virus antigen than the vaccine JE‐CV. Mice vaccinated with JE‐CV, on the other hand, only elicited a cellular immune response against the YFV NS3 protein.

In conclusion, satisfactory immune protection of viral vaccines entails both cellular and humoral immune responses. JEV nonstructural proteins elicited a robust and specific T‐cell response and conferred immune protection. The application of heterologous flavivirus as a live‐attenuated vaccine backbone is unlikely to induce an adequate T‐cell response that would target the vaccine strain virus but not backbone virus, which might reduce the efficacy of the immunization.

## 5 MATERIALS AND METHODS

### 5.1 Cells, virus, vaccine, and mice

Vero cells were cultured in Dulbecco's Modified Eagle Medium (DMEM, Invitrogen, USA) supplemented with 10% heat‐inactivated fetal bovine serum (FBS). BHK‐21 (baby hamster kidney) cells were cultured in Minimum Essential Medium (MEM, Invitrogen, USA) supplemented with 10% FBS at 37°C in a 5% CO_2_ atmosphere.

The JEV live‐attenuated vaccine SA14‐14‐2 (GenBank accession no. D90195) was procured from the Chengdu Institute of Biological Products Co., Ltd (lot: 202007A114‐1). The YFV‐17D‐based JE chimeric live‐attenuated vaccine IMOJEV (JE‐CV) was a commercial vaccine constructed with SA14‐14‐2 prM and E antigen genes using the live‐attenuated virus YFV‐17D as a backbone.^67^ JE‐CV was passaged one time in Vero cells and stored at −80°C. The virus strains YFV‐17D (GenBank accession no. FJ654700) and JEV P3 were maintained at National Institutes for Food and Drug Control (NIFDC). A standard plaque assay using BHK‐21 cells was used to determine the viral titer, and the viral stocks were stored in aliquots at −80°C.

Four‐week‐old female BALB/c mice and 17–19‐day‐old Kunming mice (12–14 g) were purchased from the Center of Animal Breeding of NIFDC. All mice were acclimated in a pathogen‐free environment.

### 5.2 Vaccination and challenge

The immunogenicity of JE‐CV and SA14‐14‐2 was tested in 17–19‐day‐old Kunming mice (12–14 g). The animals were vaccinated subcutaneously (sc) with a single dose of 10, 100, 1000, and 10,000 PFU, respectively. After 14 days (postvaccination), mice were intraperitoneally (ip) administered a lethal virus dose (3000 LD_50_) of JEV P3 strain, followed by a sham intracerebral (ic) injection with phosphate‐buffered saline (PBS). Mortality was observed and recorded daily for a duration of 14 days after the vaccination.

### 5.3 PRNT

PRNT was conducted in mice that received the 10, 100, 1000, and 10000 PFU SA14‐14‐2 or JE‐CV by the sc route. In brief, serum was collected from mice at 14 days postvaccination after eyeball blood extraction. Inactivation (by heating) of the sera was done for 30 min at 56°C, and neutralizing antibodies titer against JEV P3 strain was determined by PRNT. The sera obtained from mice injected with PBS served as negative controls. DMEM containing 2% FBS was used to prepare a 1:5 dilution of serum. Two‐fold serial dilutions of sera were mixed with equal volumes of JEV P3 in DMEM supplemented with 2% FBS to yield a mixture containing approximately 500 PFU/ml. The mixtures were incubated at 37°C for 90 min and were added to a BHK21 cell monolayer for absorption (1 h), followed by overlaying with a medium containing 1% methylcellulose with 10% FBS for 5 days. To visualize the plaques, the cells were fixed with 5% formaldehyde and stained with 0.25% crystal violet. The neutralizing antibody titer was calculated as the reciprocal of the highest dilution that resulted in a 50% reduction compared to control sera.

### 5.4 Preparation of antigen stimulations

The peptide pools consist of 15‐mer peptides that overlap by 10 amino acids, thus representing the NS3 consensus of JEV SA14‐14‐2 (GenBank accession no. MH258852) and YFV‐17D (GenBank accession no. FJ654700), which were synthesized by Shanghai Qiangyao Biotechnology Co., Ltd. The inactivated whole‐virus antigen and cell lysate total antigen for JEV SA14‐14‐2 and YFV‐17D were prepared by infecting Vero cells with JEV SA14‐14‐2 and YFV‐17D, respectively, at a multiplicity of infection (MOI) of 0.1. Cell supernatants of YFV‐17D and JEV SA14‐14‐2 post infection were used for the whole‐virus antigen stimulation, and cells were harvested by trypsinization when the cytopathic effect was visible. After centrifugation, cell pellets were resuspended in PBS, while cell lysates were prepared by four freeze–thaw cycles. UV irradiation was carried out overnight to inactivate the virus in the cell lysate preparations, and large debris were removed by filtration through 70‐μm cell strainers (Biosharp Life Sciences).

### 5.5 Mouse spleen's processing for the preparation of single‐cell suspensions

Four‐week‐old BALB/c mice were vaccinated with 10^4^ PFU of SA14‐14‐2 and JE‐CV, respectively. After 4 weeks (postvaccination), the mice were euthanized and spleens were collected for ELISPOT and flow cytometric analyses. Spleens (immersed in mouse lymphocyte separation medium [Dakewe]) were forced through 40‐μm cell strainers (FALCON) using syringe plungers to produce single‐cell suspensions. After adding 1 ml of RPMI 1640 medium, the liquid was separated and centrifuged for 30 min at 800 × *g*. After centrifugation, the middle layer of the lymphocytes was gently aspirated and placed in a centrifuge tube containing 10 ml of RPMI 1640 medium. The cells were washed upside down and then centrifuged for 10 min at 250 × *g*. Afterward, the supernatant was discarded and the cells were suspended in 1 ml of serum‐free medium (Dakewe). The viable cells were counted and the concentration of the cells was adjusted to 2.5 × 10^6^ cells/ml.

### 5.6 ELISPOT analysis

Following the manufacturer's instructions, the IFN‐γ ELISpot‐plus kit (Mabtech), including precoated plates, antibodies, and substrate, was used to perform the mouse IFN‐γ ELISPOT analysis. In brief, 2.5 × 10^5^ mouse splenocytes/well were plated with 50 μg/ml of SA14‐14‐2 or YFV‐17D NS3 peptide antigen or 100 μg/ml of total Vero cellular antigen or whole‐virus stimulations of YFV‐17D or JEV SA14‐14‐2 diluted with serum‐free medium (Dakewe). After incubation (for 24 h at 37°C), mouse IFN‐γ spots were made visible by the addition of detection antibodies, streptavidin‐HRP, and substrate. Afterward, the plates were scanned with an ImmunoSpot S6 universal reader (CTL).

### 5.7 Staining of intracellular cytokines and flow cytometric analysis

Flow cytometry was used to evaluate the frequency of production of IFN‐γ, TNF‐α, and IL‐2 by CD4^+^ or CD8^+^ cells in the vaccine and control groups. Splenocytes were isolated and seeded at 10^6^ cells/well in duplicate in serum‐free medium and stimulated for 8 h with 50 μg/ml of SA14‐14‐2 or YFV‐17D NS3 peptide antigen or with 100 μg/ml of total Vero cellular antigen at 37°C and 5% CO_2_. Also, 5 μg brefeldin A (BFA) (BioLegend, Cat# 420601) was added to block IFN‐γ, TNF‐α, and IL‐2 secretions at 6 h before the endpoint. Afterward, cells were harvested in a tube and stained with PE‐conjugated anti‐mouse CD3e, FITC‐conjugated anti‐mouse CD4, and PerCP/Cy5.5‐conjugated anti‐mouse CD8a antibodies (BioLegend, Cat# 100206, 100510, 100734), followed by 20‐min incubation in the dark. Intracellular cytokines were detected after 30‐min incubation with APC‐Cy7‐conjugated rat anti‐mouse IL‐2 (BD, Cat# 560547), APC‐conjugated anti‐mouse IFN‐γ (BioLegend, Cat# 505809), and PE/Cyanine7‐conjugated anti‐mouse TNF‐α (BioLegend, Cat# 506324) antibodies in the dark. FVS510 (BD, Cat# 564406) was used to distinguish viable cells. At least 50,000 events were analyzed with a BD LSRII flow cytometer and Summit software.

### 5.8 Statistical analysis

Statistical analysis was performed using GraphPad Prism version 9. Two‐way analysis of variance (ANOVA) with Tukey's multiple comparisons post hoc test was performed to calculate the *p*‐values between the vaccine and control groups in the ELISPOT and flow cytometric analyses. Two‐way ANOVA, followed by Tukey's multiple comparisons post hoc test, was carried out to determine the difference in the neutralization titers between the two vaccines (SA14‐14‐2 and JE‐CV). Log‐rank (Mantel–Cox) survival analysis test was performed to test for statistical significance, and *p*‐values less than 0.05 were considered statistically significant.

## FUNDING INFORMATION

Not applicable.

## CONFLICT OF INTEREST

The authors declare that there are no conflicts of interest regarding the publication of this article.

## AUTHOR CONTRIBUTIONS

Enyue Fang, Yuhua Li, and Yongxin Yu conceived and designed the experiments. Enyue Fang, Xinyu Liu, Xiaohui Liu, Ming Li, Ling Wang, Miao Li, Zelun Zhang performed the experiments. Enyue Fang analyzed the data. Enyue Fang, Xinyu Liu, Yuhua Li, and Yongxin Yu wrote the manuscript.

## ETHICS APPROVAL

All animal studies were approved by the Experimental Animal Welfare and Ethical Committee of the NIFDC, China, and were conducted according to institutional guidelines.

## Data Availability

All data are available from the corresponding authors upon request.
